# Investigation of factors affecting egg breakage resistance in laying hens using data mining and machine learning methods

**DOI:** 10.3389/fgene.2026.1898974

**Published:** 2026-08-27

**Authors:** Şenol Çelik, Turgay Şengül, Ömer Şengül

**Affiliations:** 1 Department of Animal Science, Agricultural Faculty, Bingöl University, Bingöl, Türkiye; 2 Department of Animal Science, Agricultural Faculty, Bursa Uludağ University, Bursa, Türkiye

**Keywords:** eggshell quality, eggshell strength, layer, random forest, support vector

## Abstract

**Introduction:**

This study aims to evaluate the predictive performance of different machine learning algorithms and to identify the key factors influencing eggshell breaking resistance in laying hens.

**Methods:**

Three machine learning methods, C5.0 decision tree, Random Forest (RF), and Support Vector Regression (SVR), were applied to egg classification and prediction tasks. Eggshell strength (ER) was predicted using egg weight (EW), shell weight (SW), shell thickness (ST), and shape index (SI).

**Results:**

The C5.0 decision tree, trained on 464 eggs, achieved an overall classification accuracy of 65.1%, with a tendency to misclassify brown eggs as white, suggesting potential feature overlap or class imbalance. Among the regression models, RF outperformed SVR, yielding higher R^2^ (0.852 vs. 0.553) and adjusted R^2^ (0.835 vs. 0.548) values, along with lower error metrics (MSE, RMSE, and MAPE). In addition to superior predictive accuracy, the RF model provided insights into the relative importance of egg quality traits affecting eggshell breaking resistance.

**Discussion:**

Overall, the findings indicate that ensemble-based machine learning methods are effective tools for both accurate prediction and identification of influential factors related to eggshell strength.

## 1 Introduction

Eggshell quality is very important, as only eggs with undamaged shells are considered hatching or eating eggs. In laying hen breeding, eggshell quality is an important feature taken into consideration as much as egg production and egg size. Obtaining eggs with high eggshell quality is of great importance in farms that produce eating or hatching purposes. Obtaining quality shell eggs in poultry farms means more production per laying hen and higher profitability. In eggs with insufficient shell strength, the rate of broken and cracked increases significantly. The importance of eggshell quality in egg production has increased with the automation of the production system. Petek et al. (2004) reported that the rate of broken and cracked in the eggs of chickens raised in a free-range system was significantly lower than in a cage system. Dayılar (2023) reported the broken and cracked rates of eggs produced in free range and cage systems as 2.01% and 3.5%, respectively, for the 65th week.

Consuming cracked eggs poses a risk to human health. This is an important problem for both producers and consumers. Poor quality eggshells reduce profitability and increase food waste. This harms both egg processing and farming businesses as eggs cannot be sold or have a low market value (Kemps et al., 2006). Broken and cracked eggs that occur both during the production phase (especially in the cage system) and during transportation can be sold at lower prices than their value. Using breeder eggs with poor shell quality during incubation significantly reduces hatchability. Factors affecting egg shell quality include genotype, egg size and shape, shell thickness and shell weight, production period and oviposition time. It has been reported that egg shell thickness significantly affects other properties and durability of the egg (Ketta and Tumova, 2018). In addition to eggshell durability, eggshell color profile is also an important feature in terms of consumer perception. In some countries (United Kingdom, Italy, Portugal, Ireland, Southeast Asia, Australia and New Zealand), brown eggs are generally preferred instead of white eggs (Odabaşı et al., 2007).

The eggshell is a complex structure that serves crucial biological purposes for both the growth of the chicken embryo and the preservation of the egg’s inside contents after deposition. It regulates gas and water exchanges through the pores during egg storage and incubation, ensuring a strong resistance to mechanical impacts and preventing microbial contamination (Solomon, 2010; Samiullah et al., 2014). Poultry farms are very interested in determining eggshell quality parameters that affect the eggshell’s resistance to breakage. Yang et al. (2009) reported that there was a significant relationship between eggshell color and breaking strength in chicken eggs. The eggshell, a highly organized mineralized structure, serves as a protective barrier for the eggs during incubation in addition to making egg transportation and preservation easier (Zhao et al., 2016). Several variables influence eggshell strength, including egg weight and the crystallographic direction of the mineralized eggshell (Pérez-Huerta and Dauphin, 2016; Le Roy et al., 2021). Eggshell weight is positively impacted by older laying hens, but eggshell strength and thickness are negatively impacted, leading to significant financial losses from broken eggs (Ketta and Tumova, 2016). The microstructure, thickness, specific gravity, mass, volume, surface area, and shell percentage of an egg all have an impact on the eggshell’s strength. The form of hen eggs that are laying has attracted attention among these parameters (Mónus and Barta, 2005; Nedomova et al., 2009). The width to length ratio of an egg is known as the shape index, and it's a crucial factor in assessing the quality of eggs (Sarıca and Erensayın, 2009; Duman et al., 2016). The traditional geometrical criterion used to characterize the form of an eggshell is the shape index (Sarıca and Erensayın, 2004). In addition to the shape index, other parameters and corresponding mathematical formulas have been developed to help define egg quality (Narushin, 1997).

Although numerous studies have examined eggshell quality using conventional statistical approaches, these methods often fail to capture complex and nonlinear relationships among egg traits. Moreover, studies that simultaneously compare machine learning models in terms of predictive accuracy and factor importance for eggshell breaking resistance remain limited. Therefore, the objective of this study is to apply and compare different machine learning algorithms to predict eggshell breaking resistance in laying hens and to identify the relative importance of egg quality traits, genotype, eggshell colour, and oviposition time contributing to eggshell strength.

## 2 Materials and methods

### 2.1 Materials

The material of the present study consisted of eggs obtained from laying hens belonging to two commercial genotypes: white layer (Isa Tinted) and brown layer (Lohmann Brown), reared under a free-range production system. The study was conducted on a private poultry farm with a total area of 3.5 decares, including an indoor housing area of 140 m^2^.

Hens were housed indoors during the night and allowed outdoor access for approximately 12 h during daylight. An additional 4 h of artificial lighting was provided daily. Birds were fed a commercial layer diet containing 17% crude protein and 2,750 kcal/kg metabolizable energy, with *ad libitum* access to feed and water. The outdoor area was planted with clover to allow natural grazing behaviour.

Egg collection was carried out once per week for four consecutive weeks, resulting in four sampling periods. During each sampling day, eggs were collected at four predefined time intervals: 07:30–09:30, 09:30–11:30, 11:30–13:30, and 13:30–15:30.

In total, 464 eggs were collected, comprising 234 brown-shelled and 230 white-shelled eggs. Eggs were not traceable to individual hens; therefore, all observations were treated as independent at the egg level. The potential implications of repeated sampling across weeks are addressed in the Statistical Analysis section. The following methods and equations were used to determine the quality characteristics of eggs (Nishiyama, 2012; Indarsih et al., 2021).

Egg weight (g): Measured with Digital Egg Tester.

$$\text{Shape index }\left(\%\right)=\left(\text{Egg width}/\text{Egg length}\right)\times 100.$$

The Shape Index can range between 0 and 1.0 represents a line, while 1 represents a perfect circle.

Shell weight (g): The shells of the broken eggs were measured with a 0.01 g precision scale 24 h after they were left to dry.

Shell thickness (mm): It was determined by taking the average of the measurements made from the pointed, middle and blunt parts of the shell after the egg was broken.

Breaking resistance (kg/cm^2^): Measured with Digital Egg Tester.

Permission was obtained from the private sector chicken farm where the study was conducted. Since the study was conducted in a private poultry company, we did not intervene in the routine care and feeding practices of the poultry farm. The procedures and practices related to the animals were continued by the employees of the enterprise. We only participated in the egg collection and classification works carried out at certain times of the study. Feeding and reproduction processes were not intervened, eggs were collected and analyzed at Bingöl University Animal Nutrition Laboratory.

### 2.2 Methods

#### 2.2.1 Data preprocessing

The dataset was checked for missing values and possible outliers before the model was developed. There were no missing observations found. Variance inflation factors (VIF) were used to measure multicollinearity among predictor variables; all VIF values were below the generally accepted threshold, suggesting no evidence of problematic multicollinearity. Prior to model training, all continuous predictor variables were normalized to a mean of zero and a standard deviation of one since Support Vector Regression (SVR) is sensitive to variations in variable scale.

For the SVR model, continuous predictor variables were standardized using Z-score normalization (mean = 0, standard deviation = 1). During the 10-fold cross-validation procedure, scaling parameters (mean and standard deviation) were estimated from the training subset of each fold and subsequently applied to the corresponding validation subset to prevent information leakage. Unless otherwise stated, all descriptive statistics and example predictions reported in the Results section are presented on the original measurement scale.

#### 2.2.2 C5.0 algorithm

Eggshell color was categorized using the C5.0 decision tree algorithm according to egg quality attributes. The trials parameter, which establishes the maximum number of boosting rounds, was used to incorporate boosting in order to enhance classification performance. By eliminating branches that did not significantly improve classification accuracy, automatic pruning was used to prevent overfitting and reduce model complexity (Yu et al., 2023; Xin et al., 2024).

#### 2.2.3 Random forest (RF) algorithm

Random Forest (RF) was used to predict eggshell breaking resistance because of its ability to model complex nonlinear relationships while minimizing overfitting through ensemble learning (Breiman, 2001; Cutler et al., 2012; Rushall and Sinha, 2013).

Using bootstrap samples of the training data, the method builds many decision trees, taking into account a random selection of predictor variables at each split. The average of each tree’s projections yields the final forecasts.

Out-of-bag (OOB) error estimates, which offer an unbiased assessment of prediction accuracy without requiring an independent validation dataset, were used to evaluate the model’s performance internally. The number of trees (ntree), the number of predictor variables chosen at random at each split (mtry), and the minimum node size were the main hyperparameters. On determine the relative contribution of egg quality characteristics on eggshell breaking resistance, variable significance measures were taken from the final RF model.

#### 2.2.4 Support vector regression (SVR)

An alternative machine learning method for estimating eggshell breaking resistance was Support Vector Regression (SVR) (Vapnik, 2013; Tran et al., 2024). SVR uses the ε-insensitive loss function to minimize model complexity and prediction error while estimating nonlinear relationships by applying kernel functions to translate predictor variables into a higher-dimensional feature space.

The regularization parameter (C), kernel-specific parameters, and the prediction tolerance-controlling ε parameter were adjusted to optimize the model. Predictions were produced from the optimized regression function following parameter estimates, and model training adhered to the usual optimization methodology outlined by Vapnik (2013) and Tran et al. (2024).

#### 2.2.5 Model training and validation

To reduce overfitting and ensure generalizability, all machine learning models were evaluated using k-fold cross-validation (k = 10). Data were randomly partitioned into training and validation folds, and model performance metrics were averaged across folds. For Random Forest models, cross-validation results were complemented by OOB estimates. Model performance was reported as mean ± standard deviation across folds. This approach allowed robust comparison among models and addressed concerns regarding optimistic bias.

To comparatively the predictive performances of the methods, the following model evaluation criteria were calculated (Willmott and Matsuura, 2005; Liddle, 2007; Takma et al., 2012).

Coefficient of Determination,

$$R^{2}=1-\frac{\sum_{i=1}^{n}{\left(Y_{i}-{\hat{Y}}_{i}\right)}^{2}}{\sum_{i=1}^{n}{\left(Y_{i}-\bar{Y}\right)}^{2}}$$

Adjusted Coefficient of Determination:

$${Adj.R}^{2}=1-\frac{\frac{1}{n-k-1}\sum_{i=1}^{n}{\left(Y_{i}-\hat{Y_{i}}\right)}^{2}}{\frac{1}{n-1}\sum_{i=1}^{n}{\left(Y_{i}-{\bar{Y}}_{i}\right)}^{2}}$$

Mean-square error (MSE) expressed by the following formula:

$$MSE=\frac{1}{n}\sum_{i=1}^{n}{\left(Y_{i}-{\hat{Y}}_{i}\right)}^{2}$$

Root-mean-square error (RMSE) expressed by the following formula:

$$RMSE=\sqrt{\frac{1}{n}\sum_{i=1}^{n}{\left(Y_{i}-{\hat{Y}}_{i}\right)}^{2}}$$

Mean absolute percentage error (MAPE);

$$MAPE=\frac{1}{n}\sum_{i=1}^{n}\left|\frac{Y_{i}-{\hat{Y}}_{i}}{Y_{i}}\right|.100$$

Akaike’s information criteria (AIC)

$$AIC=n\;\ln\left(\frac{{\left(Y_{i}-\hat{Y_{i}}\right)}^{2}}{n-k}\right)+2k$$

Bayesian information criteria (BIC)

$$BIC=n\;\ln\left(\frac{{\left(Y_{i}-\hat{Y_{i}}\right)}^{2}}{n-k}\right)+k\;\ln\left(n\right)$$

software was used for analyses (R Core Team: R, 2021). In these measures, n represents the number of cases in a set, k is the number of terms in the methods, $Y_{i}$ and ${\hat{Y}}_{i}$ are observed and predicted dependent variable values, respectively, of the response variable.

All analyses were conducted using R software (R Core Team: R, 2021). Machine learning models were implemented using the caret package (Kuhn, 2020) while goodness-of-fit metrics were calculated using the ehaGoF package (Eyduran, 2020). Descriptive statistics, principal component analysis (PCA), biplot visualizations, and Pearson correlation coefficients were obtained using Jamovi software (The Jamovi Project, 2022; Seol, 2023). Random seeds were set to ensure reproducibility of results.

Model performance was evaluated using 10-fold cross-validation. For the Random Forest model, out-of-bag (OOB) estimates were additionally used as an internal validation measure to reduce overfitting. Hyperparameters were tuned using grid search with 10-fold cross-validation, optimizing RMSE.

#### 2.2.6 Model tuning and reproducibility

All analyses were performed in R (version 4.4.2), and a fixed random seed (set.seed (123)) was used to ensure reproducibility. Prior to SVR modelling, all predictor variables were standardized (zero mean, unit variance).

Because individual eggs could not be linked to specific hens, observations may not have been fully independent, and this limitation should be considered when interpreting the results. Model performance was evaluated using repeated 10-fold cross-validation.

## 3 Results

Eggs from white and brown laying hens were collected at four different laying times and characteristics related to shell quality were determined. Variables such as egg breaking resistance (ER), egg weight (EW), shape index (SI), shell weight (SW) and shell thickness (ST) were used as features affecting shell quality. Descriptive statistics of the data obtained are given in Table 1.

**TABLE 1:** Descriptive statistics.

Variable	Color	Oviposition time	N	$\bar{X}$	SE	SD	Minimum	Maximum
ER	White	07:30–09:30 c	60	2.932	0.11718	0.9077	1.050	5.210
		09:30–11:30 c	60	3.138	0.09238	0.7156	1.170	4.660
		11:30–13:30 c	60	3.222	0.12300	0.9528	1.190	5.380
		13:30–15:30 bc	46	3.260	0.12586	0.8536	1.240	4.890
	Brown	07:30–09:30 c	61	3.034	0.12530	0.9787	1.170	5.250
		09:30–11:30 c	59	2.956	0.12490	0.9594	0.880	5.510
		11:30–13:30 ab	58	3.562	0.14275	1.0872	0.990	6.270
		13:30–15:30 a	60	3.761	0.11915	0.9229	1.860	6.530
EW	White	07:30–09:30 ab	60	65.50	0.88073	6.8221	54.200	87.800
		09:30–11:30 c	60	62.45	0.55048	4.2640	54.000	77.900
		11:30–13:30 c	60	62.86	0.68039	5.2703	49.700	77.980
		13:30–15:30 cd	46	62.10	0.72238	4.8994	52.400	75.600
	Brown	07:30–09:30 a	61	66.04	0.79385	6.2002	48.400	85.900
		09:30–11:30 bc	59	63.94	0.80662	6.1958	52.300	81.080
		11:30–13:30 cd	58	62.22	0.65136	4.9606	52.300	72.600
		13:30–15:30 d	60	60.43	0.69130	5.3548	47.300	72.200
SI	White	07:30–09:30 ab	60	74.46	0.35109	2.7195	65.260	81.140
		09:30–11:30 a	60	74.98	0.32514	2.5186	68.950	82.360
		11:30–13:30 a	60	74.72	0.40132	3.1086	64.860	81.420
		13:30–15:30 a	46	74.71	0.43293	2.9363	69.490	79.680
	Brown	07:30–09:30 b	61	73.40	0.48407	3.7807	64.380	80.820
		09:30–11:30 ab	59	74.57	0.66186	5.0838	67.300	104.450
		11:30–13:30 a	58	74.72	0.37134	2.8281	68.440	79.130
		13:30–15:30 ab	60	74.44	0.45150	3.4973	67.700	81.880
SW	White	07:30–09:30 ef	60	5.706	0.08449	0.6545	4.170	7.790
		09:30–11:30 f	60	5.615	0.08173	0.6331	3.370	7.770
		11:30–13:30 de	60	5.891	0.07940	0.6151	4.180	7.050
		13:30–15:30 cd	46	6.013	0.08322	0.5644	5.000	7.140
	Brown	07:30–09:30 bc	61	6.144	0.08587	0.6707	4.940	7.950
		09:30–11:30 cd	59	6.030	0.07562	0.5808	4.790	8.030
		11:30–13:30 a	58	6.452	0.09506	0.7240	4.790	7.950
		13:30–15:30 ab	60	6.264	0.08888	0.6884	4.430	8.030
ST	White	07:30–09:30 d	60	0.349	0.00332	0.0257	0.290	0.400
		09:30–11:30 cd	60	0.353	0.00378	0.0293	0.280	0.430
		11:30–13:30 bc	60	0.361	0.00422	0.0327	0.270	0.420
		13:30–15:30 b	46	0.370	0.00446	0.0303	0.310	0.430
	Brown	07:30–09:30 b	61	0.366	0.00429	0.0335	0.280	0.440
		09:30–11:30 b	59	0.367	0.00323	0.0248	0.300	0.420
		11:30–13:30 a	58	0.391	0.00441	0.0336	0.300	0.510
		13:30–15:30 a	60	0.387	0.00442	0.0342	0.300	0.480

$\bar{X}$ : Mean, SE: Standard error, SD: Standard deviation, ER: Eggshell strength, EW: Egg weight, SI: Shape index, SW: Shell weight, ST: Shell thickness.

According to Table 1, the range of eggshell strength (ER) values was 2.932–3.761 kg/cm^2^. Brown eggs laid between 13:30 and 15:30 had the highest ER value (3.761 kg/cm^2^), whereas white eggs laid between 07:30 and 09:30 had the lowest value (2.932 kg/cm^2^). Brown eggs showed a substantial rise in ER with oviposition time; eggs placed between 11:30 and 13:30 and 13:30 and 15:30 had greater values than eggs laid earlier in the day (P < 0.05). On the other hand, there were no discernible variations in the oviposition timings of white eggs.

The range of egg weight (EW) was 60.43–66.04 g. Brown eggs laid between 07:30 and 09:30 had the highest EW (66.04 g), while brown eggs laid between 13:30 and 15:30 had the lowest value (60.43 g). As oviposition time increased, there was a noticeable general decrease in egg weight, especially in brown eggs. Compared to eggs laid later in the day, early-laid eggs tended to be substantially heavier (P < 0.05).

Shape index (SI) values ranged from 73.40% to 74.98%, with comparatively little fluctuation. White eggs laid between 09:30 and 11:30 had the highest SI value (74.98%), whereas brown eggs laid between 07:30 and 09:30 had the lowest value (73.40%). Overall, most groups showed similar results, and oviposition timing had no effect on SI.

The range of shell weight (SW) was 5.615–6.452 g. Throughout the whole oviposition phase, brown eggs were consistently heavier than white eggs. Brown eggs laid between 11:30 and 13:30 had the highest SW (6.452 g), whereas white eggs laid between 09:30 and 11:30 had the lowest value (5.615 g). Shell weight frequently increased in the middle and later sections of the day (P < 0.05), indicating significant differences among oviposition periods.

The range of shell thickness (ST) values was 0.349–0.391 mm. Brown eggs laid between 11:30 and 13:30 had the thickest shells (0.391 mm), while white eggs laid between 07:30 and 09:30 had the thinnest shells (0.349 mm). In both eggshell color groups, shell thickness tended to increase with oviposition time, with brown eggs typically having thicker shells than white eggs (P < 0.05).

Overall, better shell quality characteristics, such as increased eggshell strength, weight, and thickness, were present in brown eggs produced during the latter oviposition phases. On the other hand, regardless of shell color, eggs deposited in the early morning tended to weigh more.

**FIGURE 1:**
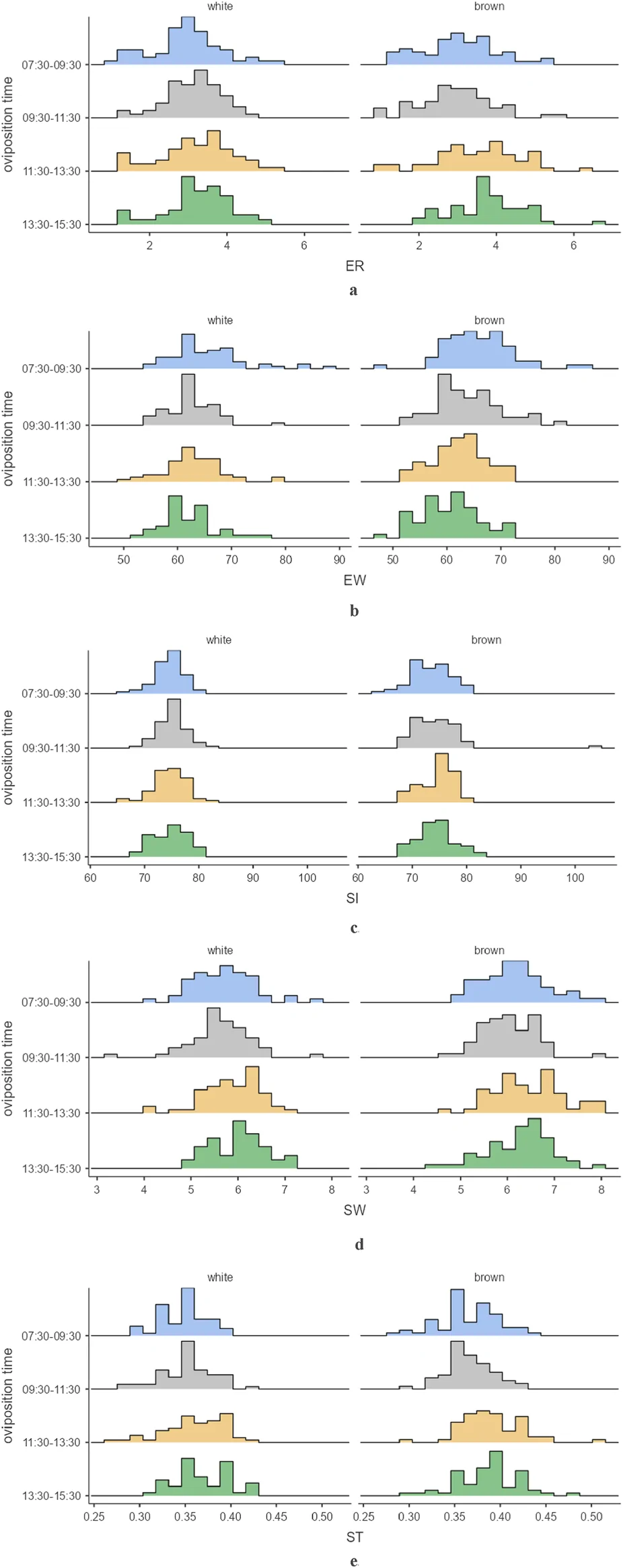
**(a)** Eggshell strength (ER). **(b)** Egg weight (EW). **(c)** Shape index (SI). **(d)** Shell weight (SW). **(e)** Shell thickness (ST).

Graphs of each egg variable in different colors and over time are presented in Figure 1.

**FIGURE 2:**
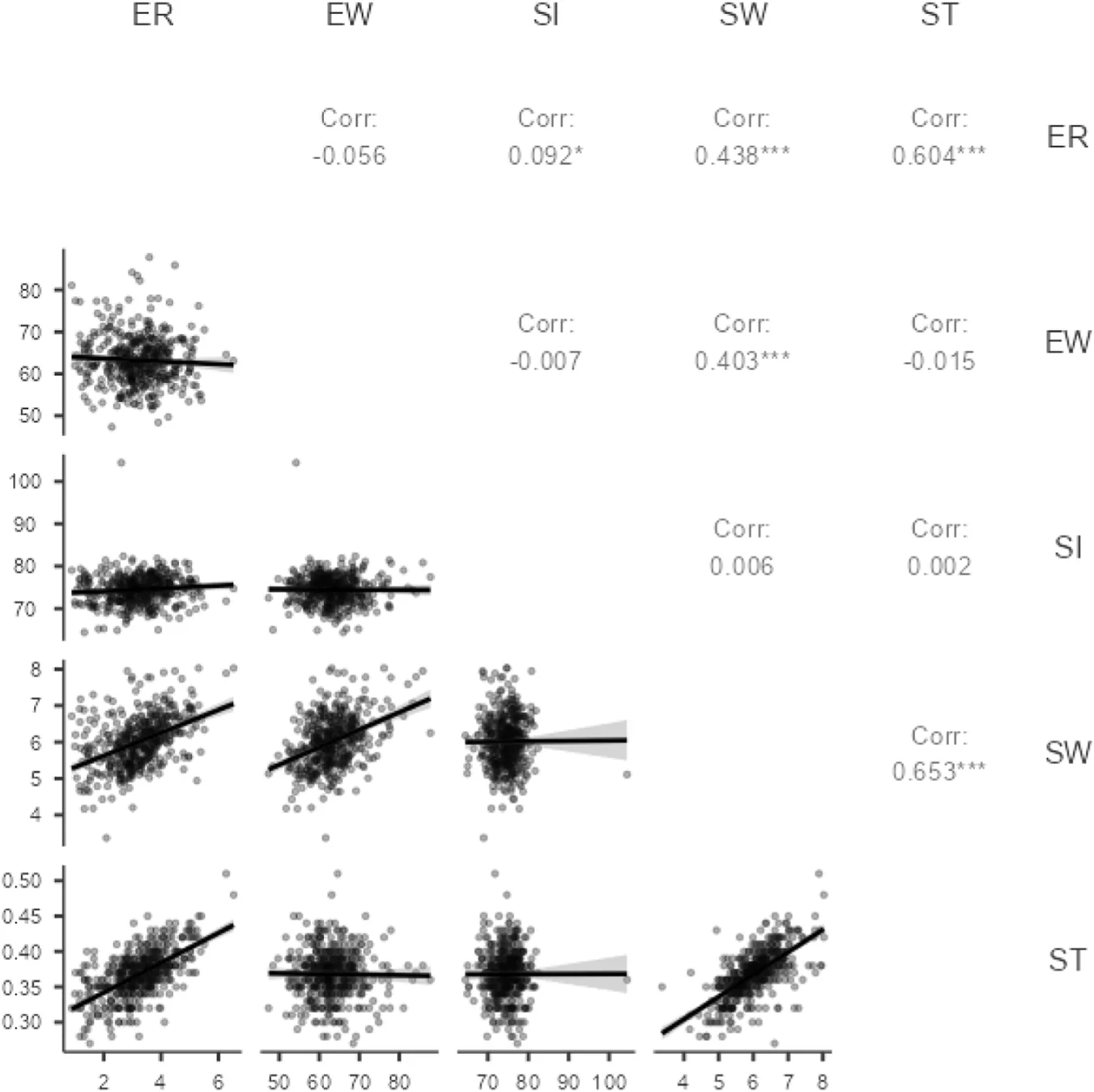
Correlation coefficients between variables. *Significant at P < 0.05, **Significant at P < 0.01, ***Significant at P < 0.001 (2-tailed test).

The correlation coefficients determining the relationships between ER, EW, SI, SW and ST variables are presented in Figure 2.

**FIGURE 3:**
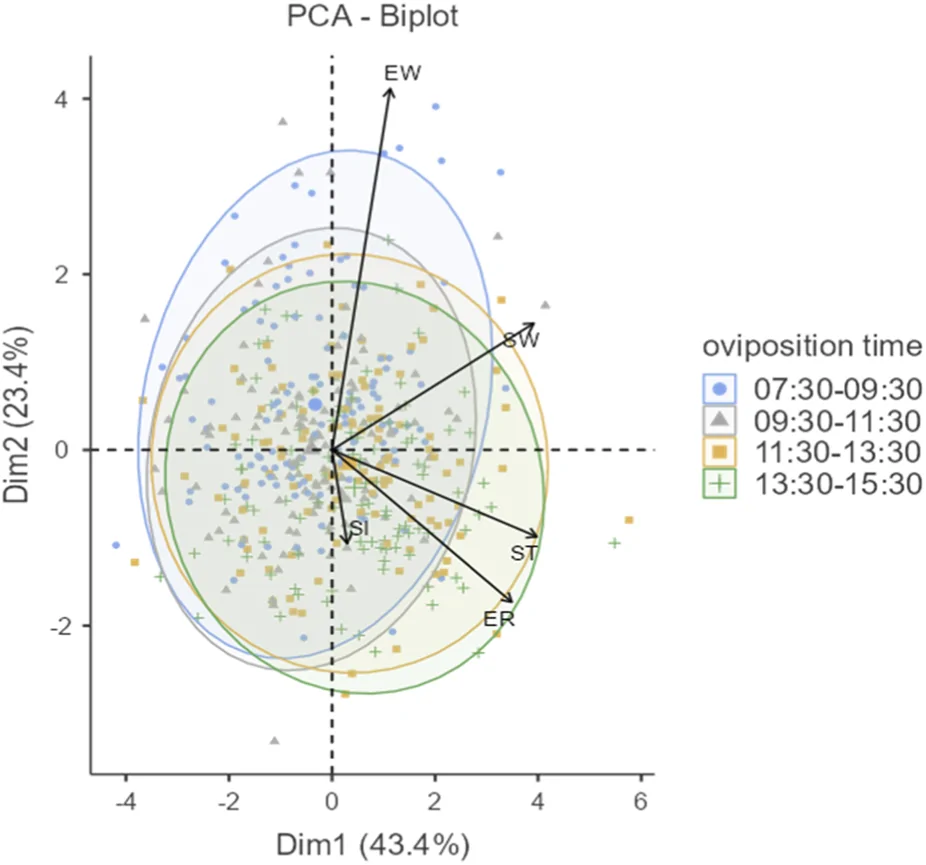
PCA-Biplot plot according to oviposition time.

**FIGURE 4:**
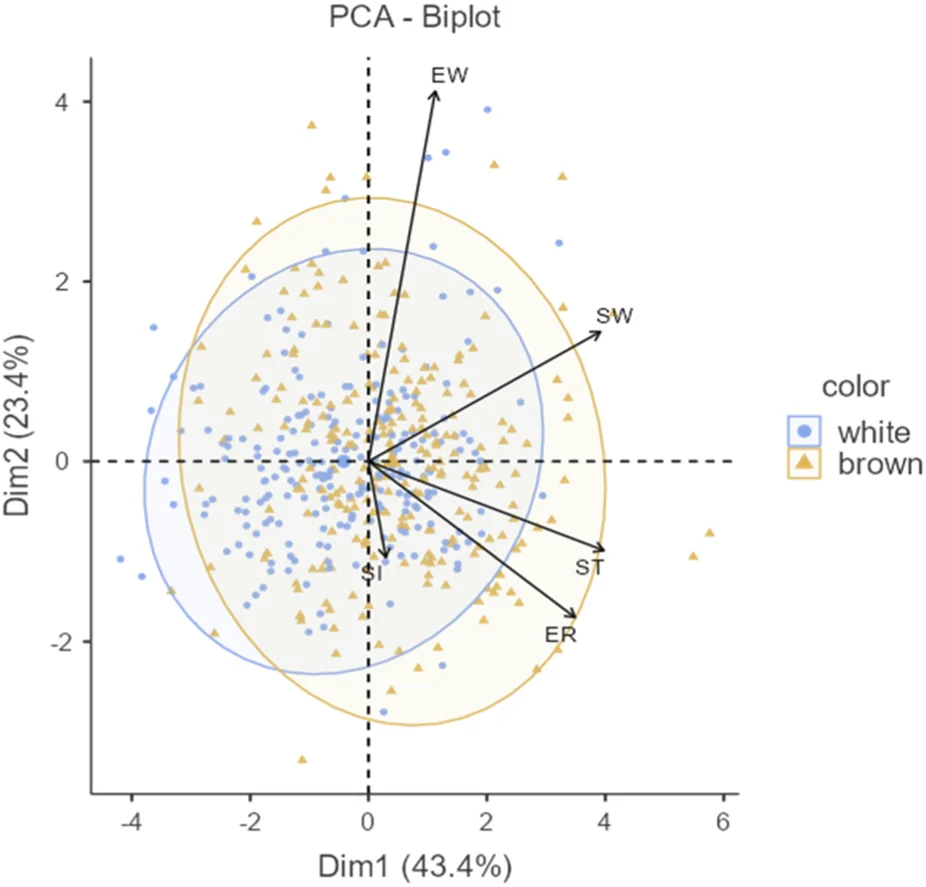
PCA-Biplot plot according to eggshell color.

The Pearson’s correlation coefficients for figuring out the connection between eggshell strength (ER) and egg characteristics are displayed in Figure 2. A significant correlation was found between ER and SI (0.092), SW (0.438) and ST (0.604), respectively. However, the correlation coefficient between ER-EW (−0.056) was found to be insignificant. A highly significant correlation coefficient of 0.653 (P < 0.001) was found between SW and ST. At the P < 0.001 level, three more highly significant correlations were found for ER-ST (0.604), ER-SW (0.438) and EW-SW (0.403). The combined representations of principal component analysis (PCA) and Biplot plots for the same variables according to laying time and shell color are presented in Figures 3, 4, respectively.

In the analysis performed with the Biplot method, PC1 (1st principal component) constituted 43.4%, PC2 (2nd principal component) constituted 23.4%, and the sum of PC1 and PC2 constituted 66.8% of the variation. Interpreting genotype-trait relationships using cutoff lines can clearly determine which genotypes are important for which traits (Karaman, 2020). The fact that the angle between the slices is between 0° and 90° is interpreted as a positive relationship with the features between this slice. An angle between 90° and 180° is interpreted as a negative relationship. If the angle is 90°, there is no relationship (Yan and Tinker, 2006; Aktaş, 2017). As the vector moves away from the origin, the variation between genotypes increases according to the trait examined, but as the vector approaches the origin, the variation between genotypes decreases (Abate et al., 2015). Accordingly, there is a positive relationship between EW and SW, and a negative relationship between EW and SI, ER and ST. A positive relationship was observed between SW and all variables. While there was a negative correlation between ST and EW, a positive correlation was observed between all other variables. There is a negative relationship between ER and EW, but a positive relationship between other variables. While there is a negative relationship between SI and EW, the relationship between other variables is positive. CART algorithm, Random Forest Algorithm and Support Vector Regression were applied to determine some egg quality characteristics that affect eggshell breakage resistance.

**FIGURE 5:**
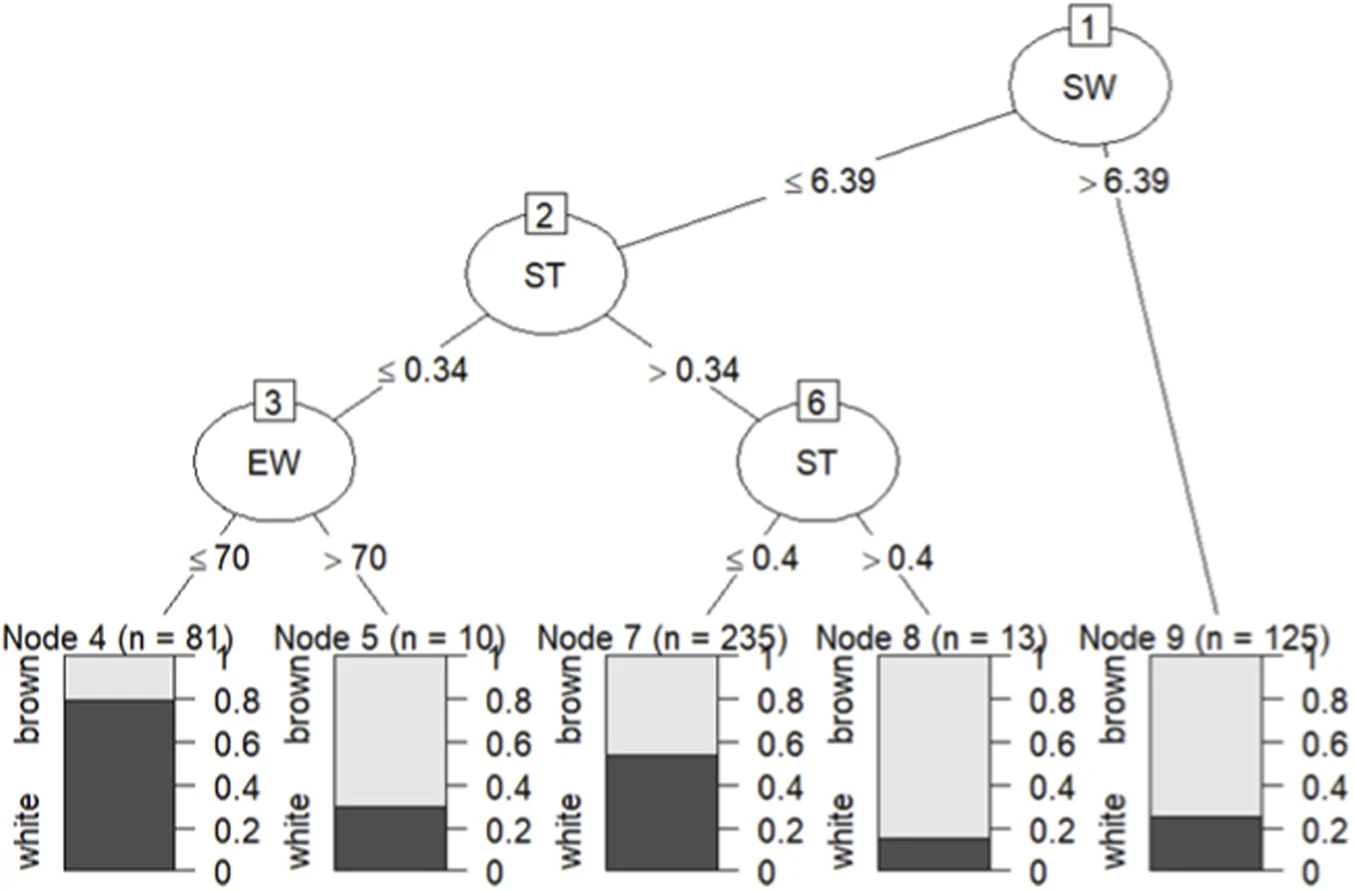
C5.0 algorithm tree diagram.

In this study, the C5.0 algorithm was employed to classify egg quality characteristics based on shell color. The C5.0 algorithm tree diagram is presented in Figure 5.

The main splitting variable found in the C5.0 classification tree was shell weight (SW), which was followed by shell thickness (ST) and egg weight (EW). Below is a summary of the primary decision-making guidelines.

Rule 1: Shell weight (primary split).

The egg is assigned to Node 9, where more than 75% of the observations are white eggs, if SW > 6.39 g.

The egg moves on to the second decision node based on shell thickness (ST) if SW ≤ 6.39 g.

Rule 2: Left subtree (SW ≤ 6.39 g).

If ST is less than 0.34 mm.

Egg weight (EW) is the next choice variable.

About 65% of the 81 eggs are categorized as brown if EW ≤ 70 g (Node 4).

Most of the ten eggs are categorized as white if EW > 70 g (Node 5).

If ST is greater than 0.34 mm.

At ST = 0.40 mm, another split is carried out.

Brown eggs are somewhat more common than white eggs among the 235 eggs if ST ≤ 0.40 mm (Node 7).

About 75% of the 13 eggs are categorized as white if ST > 0.40 mm (Node 8).

Rule 3: Correct subtree (SW > 6.39 g).

Over 75% of the 125 eggs in Node 9 are in the white eggshell class, suggesting that higher shell weight is typically connected with white eggs. Eggs with SW > 6.39 g are assigned directly to Node 9.

Example of Classification.

For instance, an egg with SW = 6.2 g and ST = 0.36 mm travels to Node 7 via the path SW < 6.39 → ST > 0.34 → ST ≤ 0.40. Given that brown eggs make up the overwhelming class at this.

The C5.0 algorithm’s Confusion Matrix is provided in Table 2.

**TABLE 2:** Confusion matrix.

Actual class	Predicted Brown (a)	Predicted white (b)
Actual Brown (a)	112	126
Actual white (b)	36	190

On the training dataset comprising 464 eggs, the decision tree model (size = 5) produced an overall error rate of 34.9%. The confusion matrix shows that out of 238 brown eggs, 112 were correctly classified and 126 were misclassified as white. For the 226 white eggs, 190 were correctly classified, while 36 were misclassified as brown (Table 2).

**FIGURE 6:**
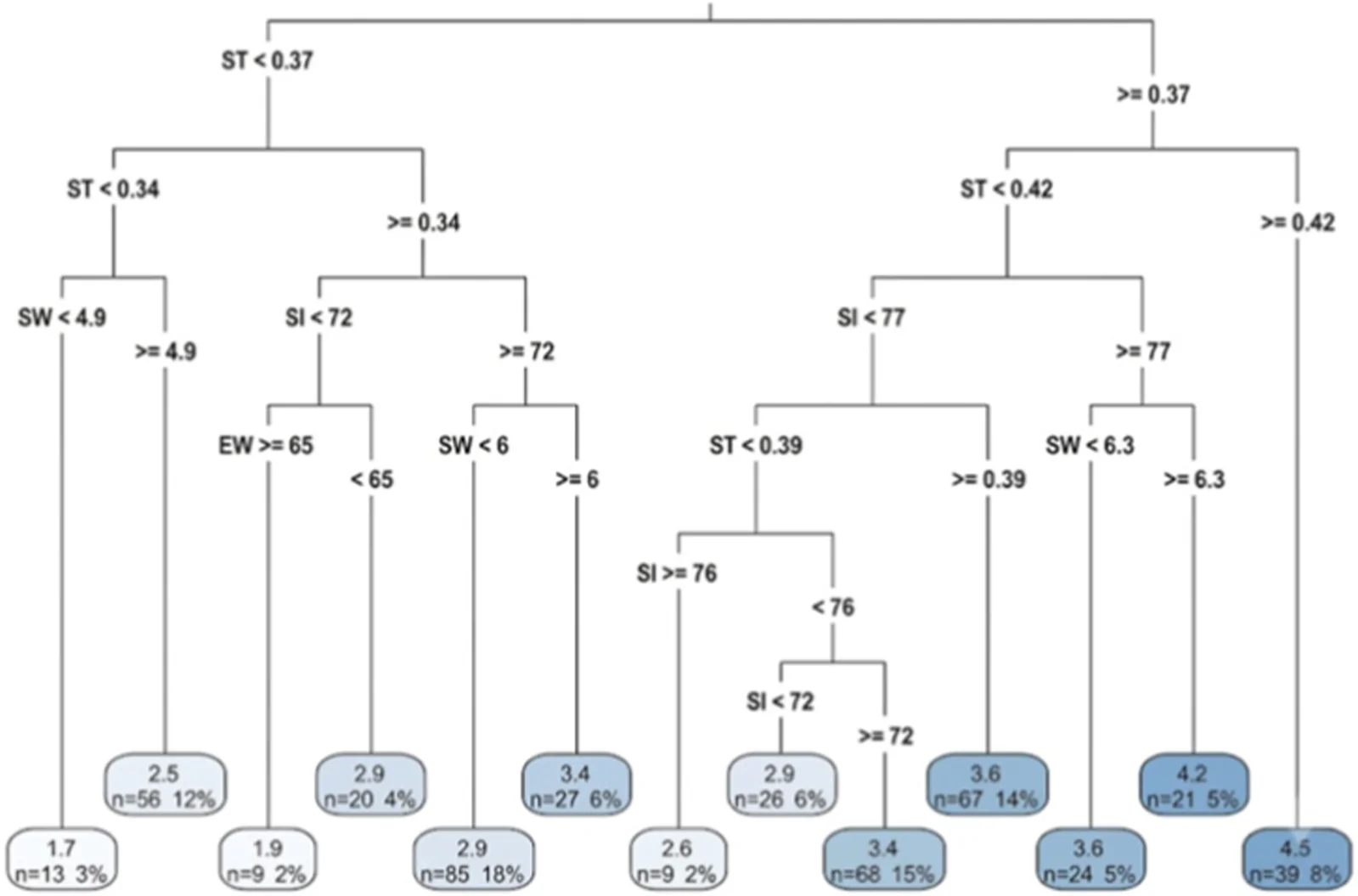
Tree structure of the Random Forest algorithm.

To determine the factors influencing eggshell breaking strength (ER), the Random Forest (RF) algorithm was applied. The resulting regression tree structure is shown in Figure 6.

The tree structure and corresponding decision rules of the Random Forest (RF) algorithm, as illustrated in Figure 6, are summarized below.

The Random Forest (RF) algorithm determined Shell Thickness (ST) to be the most significant predictor of eggshell breaking strength, as seen in Figure 6. The model’s decision tree structure showed that the explanatory variables followed a distinct hierarchical pattern. The constraint ST < 0.37 designated the root node and separated the dataset into two main subgroups according to shell thickness.

Lower shell thickness values were typically linked to lower anticipated breaking strength in the left subtree (ST < 0.37). In particular, the lowest anticipated value (1.7) was displayed by eggs with ST < 0.34 and SW < 4.9, although the predicted strength (2.5) was somewhat enhanced by increasing shell weight (SW ≥ 4.9). The relationship between Shell Index (SI) and Egg Weight (EW) became more noticeable in the middle range of ST ∈ [0.34, 0.37]: stronger shells (up to 3.4) were associated with higher SI and SW values.

On the other hand, eggs with comparatively thicker shells and gradually greater breaking strength values were represented by the right subtree (ST ≥ 0.37). For example, thicker shells (ST ≥ 0.42) produced the maximum expected strength (4.5), while combinations like ST ∈ [0.39, 0.42) and SI < 77 produced predicted values around 3.4. Likewise, increased shell resistance was positively correlated with higher Shell Weight (SW ≥ 6.3) and Shell Index (SI > 77). Overall, the RF decision tree made it abundantly evident that the main factor influencing eggshell breaking strength is shell thickness (ST), which is followed by shell weight (SW) and shell index (SI). These results demonstrate how structural and physical attributes work together to affect shell durability, highlighting how crucial it is to maintain ideal thickness and weight ratios in order to improve shell quality.

The Random Forest (RF) analysis revealed that eggs with shell thickness (ST) < 0.34 generally exhibited low egg breaking resistance (ER) values, indicating weaker shell structures. Conversely, eggs with ST ≥ 0.42 showed the highest ER value (4.5), corresponding to stronger and more durable shells. Overall, ST and shell weight (SW) were identified as the most influential variables affecting ER.

**FIGURE 7:**
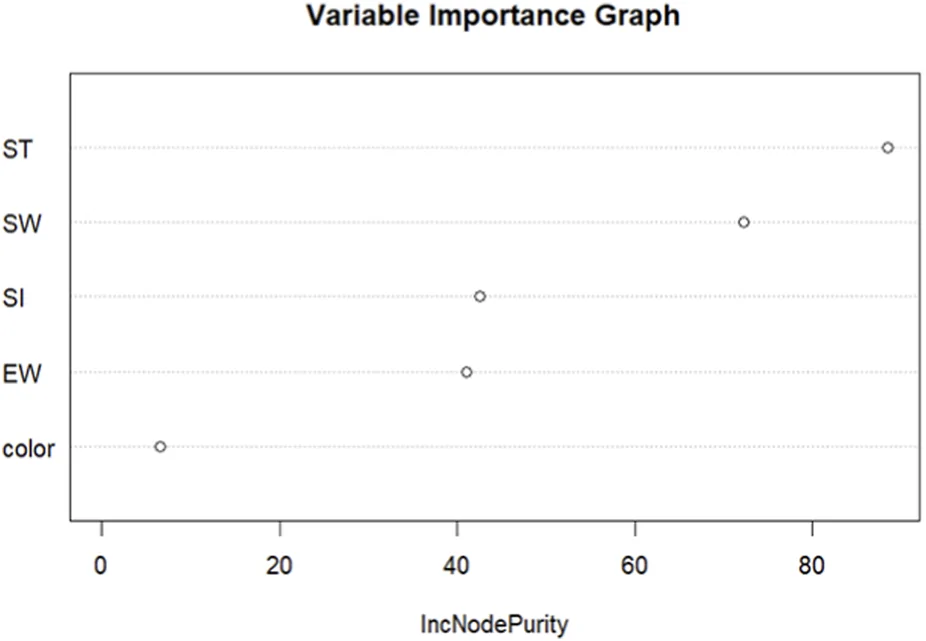
Variable importance graph in RF algorithm.

The importance of variables in the Random Forest (RF) algorithm is shown in Figure 7.

As shown in Figure 7, the Random Forest (RF) algorithm indicates that the most important variable affecting shell strength (ER) is ST. The second most important variable is SW, followed by SI as the third. EW and color are also significant contributing variables.

The coefficient of determination (R-squared) of the model is 0.852. In the Random Forest (RF) algorithm, when the values EW = 64, SI = 78, SW = 5.75, ST = 0.33, and color = ‘white’ were given, the eggshell breaking strength (ER) was predicted to be 2.711.

Another method used to predict eggshell breaking strength (ER) is Support Vector Regression (SVR). The results of the SVR algorithm are summarized as follows.

The SVR model used for predicting eggshell breaking strength (ER) is a classic epsilon-regression (eps-regression) model with a radial basis function (RBF or Gaussian) kernel, which is widely used and powerful. The cost parameter (C) was set to 10, indicating a stricter margin with less tolerance for errors. The epsilon value was 0.1, representing the tolerance range within which errors are not penalized, thereby controlling prediction accuracy. The gamma parameter, automatically set to 1/n_features, defines the spread of the kernel function. The model selected 409 support vectors, reflecting its complexity and the number of observations contributing to defining the regression function. The variable importance in the SVR algorithm is presented in Table 3.

**TABLE 3:** Variable importance graph in SVR algorithm.

Rank	Variable	Importance (loss increase)
1	SI	0.763
2	EW	0.772
3	SW	0.837
4	ST	0.914

**FIGURE 8:**
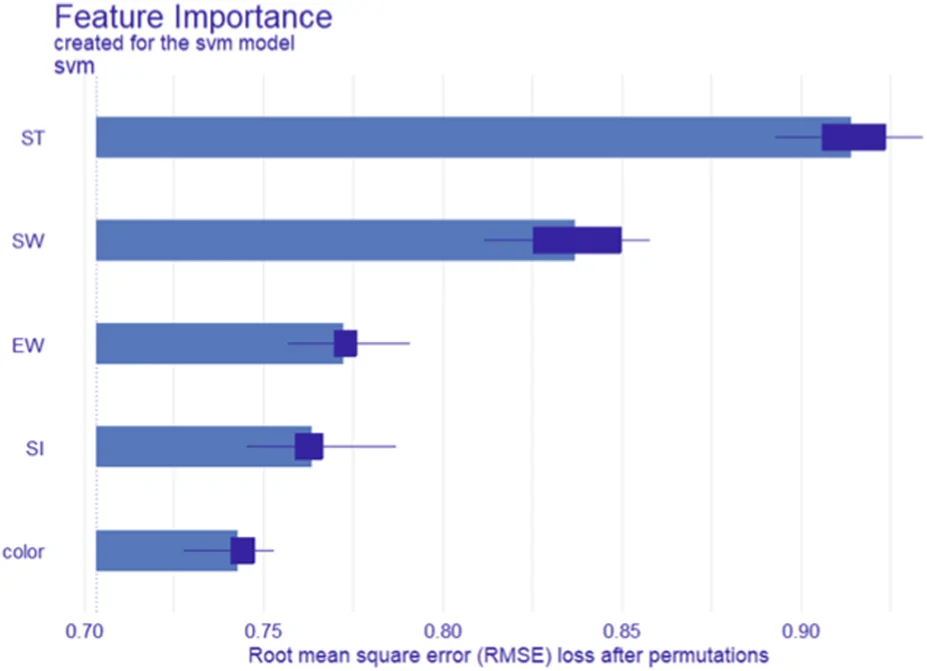
Variable importance graph in SVR algorithm.

Each variable’s contribution to the model’s performance is shown by the mean dropout loss analysis. The reference is the average loss of 0.703 for the complete model with all variables. Its efficacy is demonstrated by the fact that eliminating “color” causes the loss to grow to 0.743 and eliminating “SI” causes it to increase to 0.763. EW makes a substantial contribution (loss = 0.772), SW makes a moderate contribution (loss = 0.837), and ST is the most important variable because its removal causes the larges loss (0.914). The worst-case loss in the baseline model, which has no variables, is 1.136 (Table 3). The variable importance plot for the SVR model is shown in Figure 8.

To illustrate the predictive performance of the SVR model, an example prediction was generated using the original measurement values. For an egg with an egg weight (EW) of 2.2, shape index (SI) of 76, shell weight (SW) of 5.9, shell thickness (ST) of 0.31, and brown shell colour, the predicted eggshell breaking resistance (ER) was 3.115. Although continuous predictors were standardized during model training, the values reported here are expressed on their original measurement scale to facilitate biological interpretation. The SVR model achieved a coefficient of determination (R^2^) of 0.553. The performance statistics of the RF and SVR models are presented in Table 4.

**TABLE 4:** Model evaluation indicators.

Statistics	RF	SVR
R^2^	0.852	0.553
Adj. R^2^	0.835	0.548
MSE	0.370	0.414
RMSE	0.585	0.643
MAPE (%)	17.920	17.464

As shown in Table 4, the Random Forest (RF) model outperformed the Support Vector Regression (SVR) model in terms of overall predictive accuracy and goodness of fit. The RF model achieved higher *R* ^2^ (0.852) and Adjusted R^2^ (0.835) values, indicating a stronger explanatory power compared to the SVR model (*R* ^2^ = 0.553, Adjusted R^2^ = 0.548). Moreover, the lower MSE (0.370), RMSE (0.585), and MAPE (17.92%) values of the RF model suggest that it provides more accurate predictions.

## 4 Discussion

A prolonged duration of shell deposition in the uterus may improve shell mineralization, according to the documented increases in eggshell strength, weight, and thickness in eggs laid later in the day. On the other hand, variations in egg formation kinetics and physiological processes involved in albumen and yolk deposition may be linked to the greater egg weights observed during the early oviposition period. These results suggest that oviposition time should be taken into account when assessing egg production features since it is a significant source of variance in exterior egg quality parameters.

In the study, a positive relationship was found between egg shell resistance and shape index, shell thickness and shell weight, while a negative relationship was observed between shell breaking resistance and egg weight. This information is also confirmed in the studies of some previous researchers (Sirri et al., 2018; Wang et al., 2021; Tumová et al., 2007) reported that the differences between egg shell thickness of different genotypes were significant, but the differences between eggshell resistance were insignificant. Ketta and Tumova (2018) investigation found that eggshell strength increased with thickness. The eggshell weight was determined to be 7.23 g in the enriched cages and 5.14 g in the litter system. The Pearson correlation coefficients revealed a favorable relationship between eggshell characteristics and thickness. The scientists found that litter cages had a greater correlation (0.64, P < 0.001) than enriched cages (0.48, P < 0.001) for eggshell thickness and strength. In some studies (Aksoy et al., 2001; Tumová and Ebeid, 2005), it was reported that shell quality was better in eggs laid in the afternoon. Onbaşılar and Avcilar (2011) investigated the effects of laying time on shell quality in brown laying hens. They stated that eggs collected at 15.00 h had better shell quality than eggs collected at 09.00 h. The results obtained in this study are consistent with this information. In one study, the average breaking strength (kg/cm^2^) of chicken eggs was found to be between 1.22-2.26 (Arslan Kaya and Macit, 2018). However, in this study, the values obtained for breaking strength were found to be higher (Dama and Kaya, 2018) reported the average breaking strength of chicken eggs (kg/cm^2^) as 2.83–3.49. These results were similar to the findings of this study. In the study of (Durmuş and Türkoğlu, 2017) the average breaking resistance of hybrid eggs varied between 2.882–3.266. Al-bayati and Cufadar (2019) found the average eggshell breaking resistance of laying hens fed with different rations to be 4.64–4.86.

In this study, the correlations between eggshell strength ER and EW, SI, SW and ST were found to be −0.056, 0.092, 0.438 and 0.604, respectively. The correlation between SW and ST was 0.653, ER-ST (0.604), ER-SW (0.438) and EW-SW (0.403). These results are similar to the study of (Yang et al., 2009; Durmuş et al., 2010; Kibala et al., 2015).

The Random Forest model produced a significantly higher coefficient of determination (R^2^ = 0.852), despite the fact that the strongest individual Pearson correlation found in this study was between eggshell strength (ER) and shell thickness (ST) (r = 0.604), or roughly 36% of the explained variance. This discrepancy is to be expected since Random Forest simultaneously integrates several predictors and captures nonlinear relationships and interactions among variables, whereas Pearson correlation merely measures the linear association between two variables. As a result, the explanatory power of a single bivariate correlation cannot be directly compared to the prediction performance of the Random Forest model.

Eggshell quality traits are influenced by a number of factors, including hen breed, age, housing system, nutritional management, environmental conditions, and the measurement techniques or instruments used. Therefore, while direct comparisons with prior research are helpful for contextualizing the current findings, such comparisons should be interpreted cautiously. As a result, variations in reported eggshell strength and associated traits among research may represent both actual population differences and methodological and biological variation.

## 5 Conclusion

The following conclusions can be made in light of the C5.0, RF, and SVR models’ results:

1. C5.0 Decision Tree: Suitable for classification tasks, yet demonstrates moderate accuracy and a tendency to misclassify brown eggs as white. It could be necessary to incorporate more discriminative characteristics or balance class distributions in order to achieve further improvements.
2. Random Forest (RF): Compared to SVR, RF shows better predictive ability for continuous outcomes, offering higher goodness-of-fit and lower error metrics. Therefore, when accuracy and model generalization are important, RF is advised.
3. Support Vector Regression (SVR): Offers acceptable predictions but underperforms relative to RF, indicating sensitivity to parameter tuning and data characteristics.

Overall, it is demonstrated that ensemble learning techniques Random Forest in particular perform better in predictive tasks than both conventional decision tree and SVR approaches, underscoring their usefulness in applied agricultural and biological research contexts.

Consequently, it was determined that these statistical techniques can be effectively applied to ascertain the correlation between variables influencing eggshell strength and quality in laying hens.

## Data Availability

The original contributions presented in the study are included in the article/supplementary material, further inquiries can be directed to the corresponding author.
